# Structure, Property Optimization, and Adsorption Properties of N,N′-methylenebisacrylamide Cross-Linked Polyacrylic Acid Hydrogels under Different Curing Conditions

**DOI:** 10.3390/polym16141990

**Published:** 2024-07-11

**Authors:** Jinyu Zhang, Dezhi Qu, Shuyu Wang, Shien Qi, Huajiang Zuo

**Affiliations:** 1 College of Biological and Chemical Engineering, Guangxi University of Science and Technology, Liuzhou 545006, China; zhangjinyu9712@163.com (J.Z.); wangshuyu26@163.com (S.W.); qishien2019@163.com (S.Q.); 2 Guangxi Key Laboratory of Green Processing of Sugar Resources, College of Biological and Chemical Engineering, Guangxi University of Science and Technology, Liuzhou 545006, China

**Keywords:** pore structure analysis, adsorption performance, polyacrylic acid hydrogel, curing

## Abstract

In this study, polyacrylic acid hydrogels were prepared by modulating the cross-linking agent mass ratio using UV and heat curing methods. The structures and properties of the hydrogels were characterized and analyzed using Fourier transform infrared spectroscopy, scanning electron microscopy, and thermogravimetric analysis. The results showed that the mechanical properties of the hydrogels prepared through UV curing were better than those prepared through heat curing. The maximum mechanical tensile strength of 139 kPa was achieved at a cross-linking agent mass ratio of 3.85% with 20 min of UV curing, and the maximum mechanical compressive strength of 0.16 MPa was achieved at a cross-linking agent mass ratio of 2.91% with 20 min of UV curing. However, the hydrogels prepared by heat curing had a higher tensile strength than those prepared using the heat curing method. In addition, the thermally cured hydrogels had higher water absorption and adsorption properties. Moreover, the PAA hydrogels prepared at cross-linking agent mass ratios of 1.91 and 2.91% with 2 h of the heat curing method had the best swelling properties. Moreover, the increase in the cross-linker mass concentration led to a decrease in the pore size and porosity and to a more compact structure.

## 1. Introduction

Hydrogels are three-dimensional polymer networks containing large amounts of water. Owing to their unique networks, they have been extensively studied for various applications, such as those in the biomedical field and environmental science [1]. Acrylic acid (or acrylate) and acrylamide are widely used as monomers in hydrogels, and they have good biocompatibility and tunable physicochemical properties, making them promising hydrogel materials [2,3].

The continuous advancement in science and technology has led to deeper research on hydrogels. Among them, polyacrylic acid hydrogel, which is synthesized from acrylic acid as a monomer, has become a commonly used hydrogel, achieving significant results in various fields. For example, in environmental protection, polyacrylic acid hydrogels can adsorb and immobilize organic matter, heavy metals, and other pollutants in wastewater, thereby purifying the water. For example, Gokmen et al. [4] produced polyacrylic acid hydrogel as an adsorbent to remove certain divalent ions and trivalent iron ions in water and achieved a significant result. In addition, polyacrylic acid hydrogels can be used for sludge treatment and solidification in wastewater treatment plants. In agriculture, polyacrylic acid hydrogels can be used to retain soil water, improve soil structure, and increase water use efficiency for plant growth. For example, Yan et al. [5] prepared a slow-release fertilizer hydrogel containing nitrogen (N) and phosphorus (P) by adding ammonium chloride and potassium phosphate to acrylic acid as the monomer. In the medical field, polyacrylic acid hydrogel can also be used as a moist dressing [6] to facilitate wound healing and maintain a moist environment. Tan et al. [7] used polyacrylic acid, branched polyacrylamide, and quaternary ammonium chitosan to prepare a self-gelatinizing hemostatic powder. This prepared powder can rapidly form a hydrogel when it comes into contact with water. Additionally, when the powder is applied to wounds, it can rapidly aggregate blood cells and platelets and adsorb blood, thereby achieving hemostasis.

At present, both heat curing and light curing are the most commonly used methods to prepare polyacrylic acid hydrogels. Liao et al. [8] used static aqueous solution polymerization to prepare polyacrylic acid. Heat curing was used to prepare the hydrogels by heating the polyacrylic acid sol in a water bath at 80 °C to cure. This process allowed the hydrogel to bond with the foam support to form a porous network structure for catalytic support. Tan et al. [7] used static aqueous solution polymerization to prepare a sol. They prepared the hydrogel by adding a cross-linking agent to the sol and then cured it at room temperature in static mode. Although this is a type of thermal curing, the process takes 24 h, which is excessively long. Liang et al. [9] used a two-step cross-linking method to synthesize the polymer sol. The process involved stirring the sol at room temperature, followed by ultrasonication, and then curing it for 12 h in an oven at a temperature lower than 50 °C. Reza et al. [10] prepared nanocomposite hydrogels that were similarly cross-linked and polymerized at low or even room temperature. This modification and heating process may affect some materials, disrupt their structure, and affect their properties. In contrast, light curing is the curing method that involves exposing hydrogels to light. The main advantage of this method is that it does not require an additional heat source, thus preventing thermal damage to the hydrogel. Light curing can usually be performed at room temperature, making this method more flexible. Zhou et al. [11] developed a composite hydrogel by chemically modifying chitosan and filaggrin proteins in the presence of a photoinitiator. Qi et al. [12] designed and synthesized a photo-crosslinked injectable filaggrin hydrogel by introducing methacrylic acid groups on the side groups of filaggrin proteins. Then, the resulting products were rapidly cross-linked in situ by UV irradiation in the presence of photoinitiators to form filaggrin hydrogels as a biomimetic ECM. This method enables therapeutic treatments through minimally invasive delivery. However, the light curing method requires a specific light source, such as UV or laser light, which may limit its use in some applications. In addition, some materials may be sensitive to certain light and require some adjustments to achieve the desired result.

In this study, PAA hydrogels were prepared with an MBA cross-linker using UV curing and heat curing. This study aimed to investigate the effect of the MBA cross-linker content on the structure and properties of hydrogels and the adsorption effect on heavy metals. Additionally, the effects of the curing modes (heat and ultraviolet) on polyacrylic acid hydrogels were examined. The curing mode and cross-linking agent mass ratio significantly affected the mechanical, water absorption, and adsorption properties of the hydrogels. This study provides insight into the modification of hydrogels, enhancing their properties to expand their applications in various fields.

## 2. Materials and Methods

### 2.1. Materials

Ammonium persulfate (APS), sodium dodecyl sulfate (SDS), acrylic acid (AA), N,N′-methylenebisacrylamide (MBA), and 2-hydroxy-4′-(2-hydroxyethoxy)-2-methylpropiophenone (Irgacure I-2959) were 98% analytically pure and were purchased from Shanghai Aladdin Co., Ltd. (Shanghai, China). Lead nitrate (Pb(NO)_3_) was 98% analytically pure and purchased from Xilong Chemical Co., Ltd. (Shantou, China). Nitric acid (HNO_3_) was 65–68% analytically pure and purchased from Shanghai Aladdin Co., Ltd. (Shanghai, China). Deionized water was used in all experimental stages. All reagents were used directly without any purification.

### 2.2. Synthesis of Poly (AA)-Based Hydrogels

**Figure 1:**
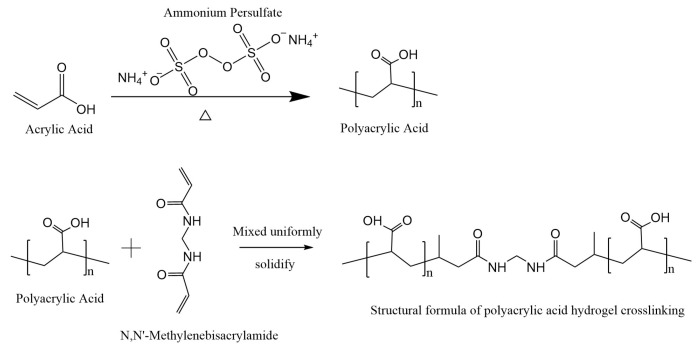
Acrylic acid polymerization and cross-linking reaction formulae.

**Figure 2:**
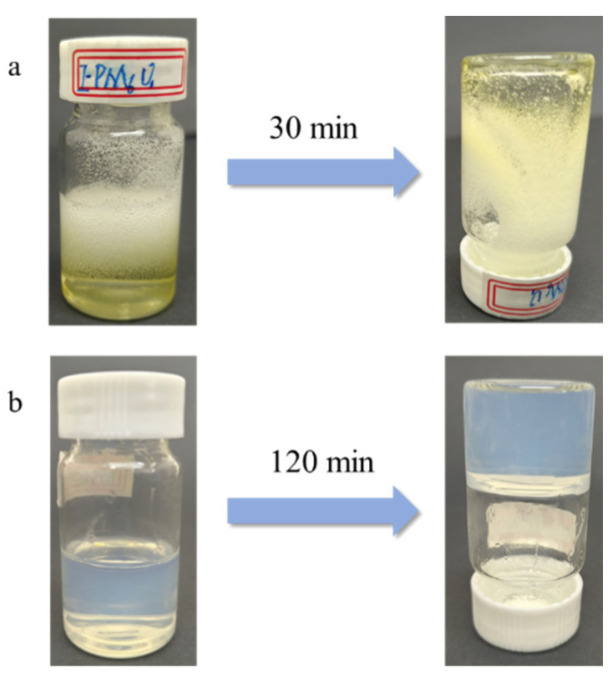
The PAA hydrogel display. ((**a**) Cross-linking effect observed after the addition of a photoinitiator; (**b**) cross-linking effect observed without the use of a photoinitiator).

Appropriate amounts of APS, SDS, and deionized water were homogeneously mixed in a four-necked flask and then placed into a water bath at 80 °C for 10 min. During this process, nitrogen was passed into the four-necked flask to remove the oxygen in the reactants and prevent AA from oxidizing in the polymerization process. Afterward, a polyacrylic acid (PAA) resin emulsion was prepared using a one-pot method, where the masses of APS and SDS were 0.75 wt.% and 2.0 wt.% of that of monomer AA, respectively. The specific reaction equation is shown in Figure 1. As shown in Figure 2, the PAA hydrogel synthesized using Irgacure I-2959 (Guzhen Jigu Co,. Ltd. Zhongshan, China) and N,N′-methylenebisacrylamide (MBA) as cross-linking agents was yellow after it was subjected to UV curing for 20 min and was not successfully cured. The PAA hydrogel prepared using an MBA cross-linking agent after UV curing for 20 min was transparent and cured successfully. Therefore, the addition of a combination of Irgacure I-2959 and a cross-linking agent did not cure the PAA resin into a hydrogel. Afterward, the MBA was used as a cross-linking agent. The same mass of PAA resin was placed into a beaker, MBA was added, and the mixture was quickly and evenly stirred. The air bubbles inside the resin were removed from the mixture by shaking it using the KQ 3200 E ultrasonic cleaner from Kunshan Ultrasonic Instrument Co., Ltd. (Kunshan, China). The PAA hydrogel was obtained after the sample was subjected to UV curing or heat curing for different durations. The wavelength of UV used for curing was 365 nm, the intensity of light was 675 Mw, and the temperature used for heat curing was 80 °C. During this process, the covalent bonding of PAA transformed the PAA chain from a linear structure to a three-dimensional mesh structure. Afterward, the PAA hydrogels were removed, rinsed, and soaked several times with deionized water to remove unreacted monomers and water-soluble impurities. The prepared hydrogels were then post-treated in three ways: first, they were freeze-dried to obtain a dry gel, followed by drying in a constant temperature oven at 80 °C until constant weight was achieved. Finally, the samples were soaked in water and preserved. The hydrogels were named PMxUy/PmxHy, where P stands for polyacrylic acid resin emulsion, M represents the type of cross-linker, x is the specific cross-linker used, U refers to the UV curing mode, H refers to the heat curing mode, and y represents the curing time, which is listed in Table 1. The cross-linking agent mass ratio was then calculated as follows (1):

(1) $$\mathrm{Cross-linking}\;\mathrm{agent}\;\mathrm{mass}\;\mathrm{ratio}\;=\frac{m_{\mathrm{MBA}}}{m_{\mathrm{total}}}\times 100\%$$

where $m_{\mathrm{MBA}}$ is the mass of the cross-linker (g), and $m_{\mathrm{total}}$ is the total mass.

### 2.3. Characterization of PAA Hydrogel

The hydrogel samples were converted into dry gels using a freeze dryer (Lab-1A-50, Poyikang, Beijing, China). Fourier transform infrared (FTIR) (Nexus-670, Nicolet, Madison, WI, USA) was used to examine the resin and dried PAA gel. The scanning range was 4000–500 cm^−1^ with a resolution of 4 cm^−1^, and 32 scans were performed. The chemical structure of the PAA hydrogel obtained from an acrylic monomer through free radical polymerization was confirmed using a Fourier transform infrared spectrometer (FTIR, Nicolet Instruments, Madison, WI, USA). The thermal stability of the PAA dry gels was tested using a synchronous thermal analyzer (TGA) (STA 449F5, Netzsch, Selb, Germany). The temperature range was 30–800 °C with a heating rate of 10 K/min. The gel samples were fractured in a liquid nitrogen environment and then mounted on the stage with conductive adhesive. Then, the samples were sprayed with gold at 15 kV for 20 s and examined using a scanning electron microscope [SEM, Phenom ProX SEM from Fuhner Scientific Instruments (Shanghai) Co., Ltd., Shanghai, China] at 10 kV voltage under standard beam conditions. The gel samples were degassed and processed at 100 °C for 8 h using a Gemini VII 2390 rapid specific surface area and porosity analyzer (BET) from McMurray Tic (Shanghai) Instruments Co., Ltd. (Shanghai, China). The BET method was used to determine the pore size distribution, while the BJH model was used to determine the pore size of the hydrogels.

### 2.4. Measurement of Solubility, Water Loss Rate, and Hygroscopicity

The prepared dry gel sample (*W*_0_, g) was first weighed and then placed into deionized water to ensure it was completely submerged. At this point, pH = 7. After a set time, the hydrogel was removed, and excess solvent was gently removed from the surface with filter paper. Then, the mass (*W*_1_, g) of the sample was quickly measured again. The solubilization ratio (*SR*) was calculated using Equation (2):

(2) $$SR=\frac{W_{1}-W_{0}}{W_{0}}\times 100\%$$

To conduct a water loss rate test, a portion of the hydrogel was first weighed and recorded as mass *W*_2_ (g). Then, the hydrogel was placed in a constant temperature chamber (BPS, Yihengkeji, Shanghai, China) at various humidities for 24 h and a constant temperature of 25 °C. After 24 h, the hydrogel was removed from the chamber, and its wet mass was measured, noted as *W*_3_ (g). Finally, the *water loss rate* was calculated using Equation (3):

(3) $$water\;loss\;rate=\frac{W_{2}-W_{3}}{W_{2}}\times 100\%$$

Determination of moisture absorption rate: To determine the moisture absorption rate of PAA hydrogel, the sample was dried to a constant weight in an oven and weighed (*W*_4_, g). The samples were placed in a constant temperature and humidity chamber for 24 h at a constant temperature at 25 °C. After equilibrium was reached, the wet mass of the hydrogel was measured (*W*_5_, g). The moisture absorption rate was calculated using Equation (4):

(4) $$\mathrm{Hygroscopicity}=\frac{W_{5}-W_{4}}{W_{5}}\times 100\%$$

### 2.5. Mechanical Testing of Hydrogels

The PAA hydrogel was transformed into a long strip specimen 20 mm in length, 10 mm in width, and 1 mm in thickness using PTFE sheets. The hydrogel was tested using an electronic universal testing machine at 25 °C and standard atmospheric pressure with a fixture of 10 mm and a rate of 20 mm/min according to the standard. The maximum load of the testing machine was 2 kN. The strength of the sample was calculated using Equation (5) [13], where w and t are the initial width and thickness of the sample, respectively. The elongation at break is the ratio of the change in tensile length of the sample to the initial mark. Each hydrogel was tested at least five times or more and averaged to minimize random errors. The modulus of elasticity was calculated using stress and strain data between 5% and 15%. The PAA hydrogels were fabricated into cylindrical specimens at a height of 10 mm and a cross-section of 30 × 10 mm using a beaker. The hydrogels were tested using mechanical compression at 25 °C and standard atmospheric pressure using an electronic universal testing machine at a compression speed of 10 mm/min in accordance with the GB/T 16491-2022 standard [14]. The maximum capacity of the testing machine was 2 kN, and the mechanical compression strength was calculated using Equation (6) [13], where s is the cross-sectional area of the specimen. The compressive strain is the ratio of the change in tensile length of the sample to the initial mark. Each hydrogel was tested at least five times, and the average value was taken to minimize random errors.

(5) $$\sigma =\frac{\mathrm{Load}}{w\times t}$$

(6) $$б=\frac{\mathrm{Load}}{s}$$

### 2.6. Applied Research on the Adsorption of Lead Ions

First, lead nitrate solutions of 0.50, 1.00, 2.00, 4.00, and 5.00 mg/L concentrations were prepared by diluting the original solution of 1000 mg/L. The adsorption values were measured using inductively coupled plasma emission spectrometry (ICP-OES), and a standard curve for Pb^2+^ was plotted. To prepare 1000 mL of Pb^2+^ at a concentration of 207.2 mg/L, 10 mL of Pb^2+^ was pipetted into a weighing flask containing 1.00 g of dry gel. The flask was uniformly shaken using a thermostatic water bath oscillator for 24 h at 25 °C and standard atmospheric pressure to allow the dry gel to adsorb Pb^2+^ in the weighing flask. The pre- and post-adsorption solutions were diluted 100 times, with their pH = 1. Experiments were performed using a 2% nitric acid solution for volume fixation. Subsequently, the adsorption values were measured using ICP-OES. The adsorption capacity was calculated using Equation (7):

(7) $$AC=\frac{C_{0}-C_{e}}{C_{0}}\times 100\%$$

where *C*_0_ is the initial concentration of heavy metal ions in the solution before adsorption, in ppm; *C_e_* is the residual concentration of heavy metal ions in the solution after adsorption, in ppm.

## 3. Results

### 3.1. FTIR Analysis

**Figure 3:**
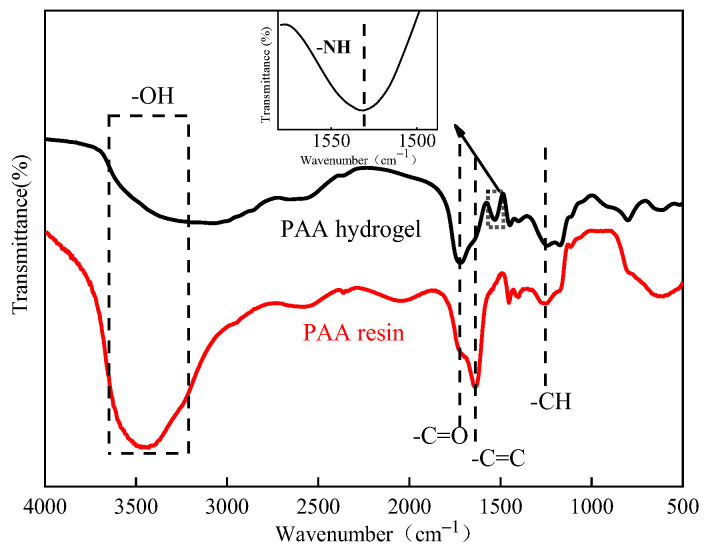
FTIR spectra of the mixture before and after curing.

The FI-IR of the PAA hydrogel and PAA resin are shown in Figure 3. The results showed that the peak positions of the two spectra are basically the same. The main characteristic peak of -COOH was located at 3452, corresponding to the -OH stretching vibration peak. The spectrogram of the PAA hydrogel displayed a weak -OH absorption peak. This peak was attributed to the increased cross-linking in the main structure of the PAA hydrogel, which restricts the movement of the carboxyl groups. This restriction reduces the likelihood of the carboxyl groups forming a hydrogen bond, resulting in a lower peak height. The -CH bending vibrational peak located at 1369 cm^−1^ indicates that the reaction of the PAA resin prepared through the emulsion polymerization of AA is almost complete. The PAA hydrogel showed a strong -C=O stretching vibration peak at 1721 cm^−1^, and the PAA resin showed a strong -C=C vibration peak at 1631 cm^−1^. In addition, the absorption peak of the PAA hydrogel located at 1531 cm^−1^ was attributed to the -NH bending vibration. No vibration peak was observed for the PAA resin, indicating that the free radical polymerization reaction of the PAA hydrogel was successful, and the MBA cross-linking agent was successfully introduced into PAA during the curing process to form the PAA hydrogel.

### 3.2. Nitrogen Isothermal Adsorption–Desorption Analysis (BET) and Pore Structure Analysis

**Figure 4:**
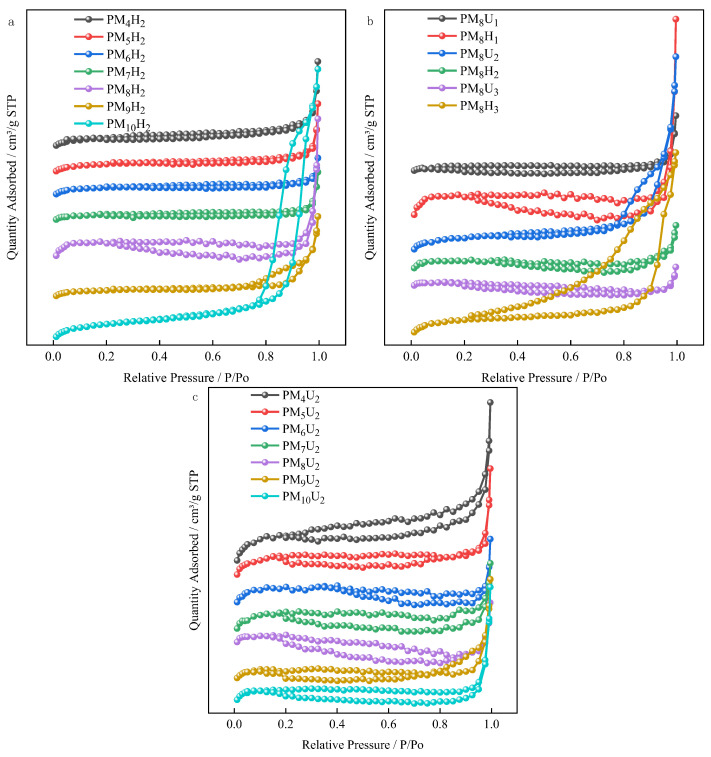
BET plots of PAA hydrogels. ((**a**) Hydrogels cured by heat for 4 h at different cross-linking agent mass ratios; (**b**) hydrogels cured by UV for 20 min at 3.85% cross-linking agent mass ratio; (**c**) hydrogels cured by UV for 20 min at different cross-linking agent mass ratios).

**Figure 5:**
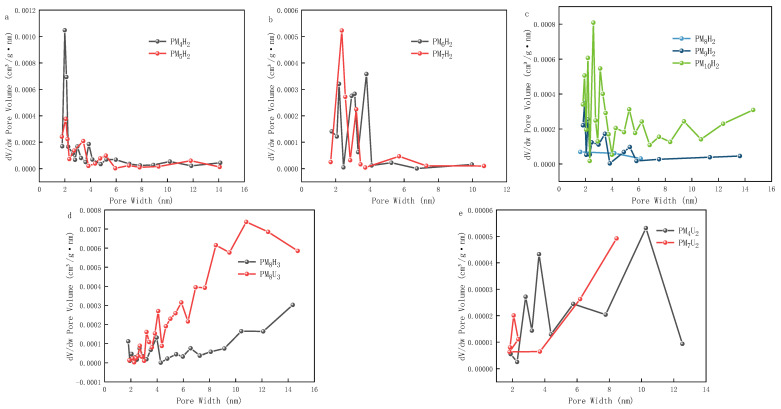
Pore size plots of PAA hydrogels. ((**a**–**c**) are hydrogels cured with heat curing for 4 h at different cross-linking agent mass ratios; (**d**). hydrogels cured with heat curing for 6 h and UV curing for 30 min at a cross-linking agent mass ratio of 3.85%; (**e**). hydrogels cured with UV curing for 20 min at different cross-linking agent mass ratios).

As shown in Figure 4a,b, the hysteresis loops of the PAA hydrogels formed using heat curing are small or almost absent, which means that the pore structure in the hydrogel is irregular, dispersed, or has small pore sizes. As shown in Figure 5a–c, more small pore sizes were formed. The increase in the cross-linking agent mass ratio led to an increase in the pore size and an uneven pore size distribution. Smaller hysteresis loops imply the poor connectivity of the pores or a narrower pore size distribution, which may have some effect on the adsorption and transport properties of the substances [15,16]. As shown in Figure 4, both the PAA hydrogels cured by heat and UV show representative III curves, which essentially have hysteresis loops and upward tailing loops. This observation indicates that the pore structure in the hydrogel consists of macropores or macropores coexist with mesopores. In this case, the large size of the pores facilitates the movement of nitrogen molecules to the pores, thus exhibiting unrestricted adsorption [17]. Non-restricted adsorption refers to the situation where the interaction between the adsorbent and the adsorbate during adsorption is not restricted by the size or shape of the pores [18]. Figure 4 shows that the isotherms continue to increase at higher relative pressures (close to 1), indicating that the adsorbent can still enter the pores or adsorb on the surface of the adsorbent even at higher pressures. The PAA hydrogel serves as an adsorbent owing to its numerous carboxy functional groups, which can interact with a variety of ions or molecules, thus achieving the adsorption of specific substances. As shown in Figure 4b,c, the PAA hydrogels formed through UV curing have large hysteresis loops, which implies the presence of regular, uniform, and interconnected mesoporous structures in the hydrogels. Large hysteresis loops usually indicate that these mesopores have high connectivity and large pore sizes, which are favorable for the transport and adsorption of substances in them [19]. As shown in Figure 5d,e, the pore size of the UV-cured hydrogels also increases with the cross-linking agent mass ratio. As shown in Figure 4b–c, the hysteresis loop conforms to the H3-type hysteresis loop line, indicating that the interior of such hydrogels mainly comprises pores of flat slit-like, crack-like, or wedge-shaped structures, which are also seen in aggregates of a laminar structure. These structures may be attributed to the stacking of lamellar particles or the formation of non-rigid aggregates. The large hysteresis loop of PM_8_H_3_ in Figure 4b indicates that the hydrogel is characterized by the presence of large pores, as shown in Figure 5d. Therefore, the occurrence of unrestricted adsorption makes the adsorbent less selective to the target substance, resulting in a lower adsorption capacity.

### 3.3. SEM Analysis

**Figure 6:**
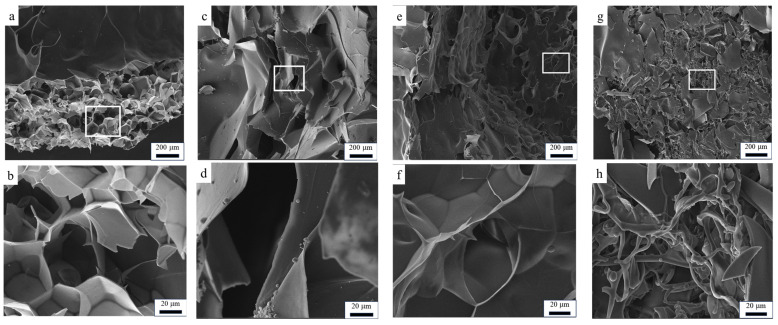
SEM images of PAA hydrogels. ((**a**) PM_9_U_2_; (**b**) partial enlargement of (**a**); (**c**) PM_9_H_2_; (**d**) partial enlargement of (**c**); (**e**) PM_8_U_2_; (**f**) partial enlargement of (**e**); (**g**) PM_6_H_2_; (**h**) local enlargement of (**g**)).

The SEM plots reveal the internal structure of the hydrogels in the lyophilized state. From the PAA hydrogel cross-section shown in Figure 6a,b,e,f, the hydrogel cured using UV has a relatively dense network structure, with most of the pores being more regular and uniform. Meanwhile, the network of the hydrogel is relatively uniform and neat, which could be attributed to the rapid polymerization process that ensured a uniform network structure [20]. As shown in Figure 6c,d,g,h, the increase in the cross-linking agent mass ratio improves the connectivity of the pores. In addition, the pore structure in the thermally cured hydrogel was more irregular and dispersed, with large and small pore diameters. Additionally, the pore connectivity of thermally cured hydrogel was poor, characterized by narrower pore distributions and particle buildup on the pore surfaces. Consequently, these factors may lead to a reduced adsorption capacity and unrestricted adsorption behavior in the hydrogel.

### 3.4. Thermal Stability Testing

**Figure 7:**
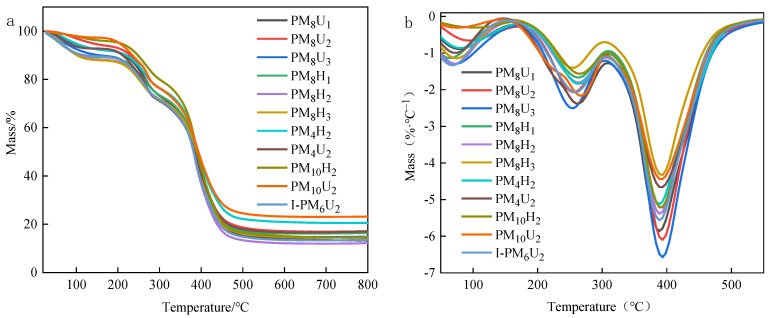
Thermal properties of hydrogels under different conditions. ((**a**) TG diagram of PAA hydrogel; (**b**) DTG diagram of PAA hydrogel).

Thermogravimetric analysis (TG) and differential thermogravimetric analysis (DTG) are methods for assessing the thermal stability of materials. It involves measuring the mass of a sample as a function of the experimental temperature under controlled conditions and different atmospheres. Figure 7 shows that the thermal decomposition process of PAA hydrogels cured under different conditions is similar and can be divided into three stages. In the first stage, the weight loss of the hydrogel occurred at approximately 100 °C. In this stage, the weight loss of the sample caused by water evaporation was minimal, indicating that the hydrogel was physically stable and maintained its structural integrity at a certain temperature. As shown in Table 2, the temperature at which 10% mass loss occurred was 100–200 °C. In the second stage, the thermal decomposition process occurred at 200–400 °C, when the hydrogel underwent initial pyrolysis, which was mainly due to the incomplete cross-linking of the branched part of the hydrogel. As shown in Table 2, the temperature at which 50% mass loss occurred was 380–390 °C. In this stage, the mass loss caused by thermal degradation mainly originated from the main body of the cross-linked hydrogel. The maximum decomposition rate of the hydrogel appeared at 387.8 °C. The difference in the initial thermal degradation temperature of the PAA hydrogels produced at different cross-linking agent mass ratios was related to the curing method. Specifically, the initial thermal degradation temperature was higher for the thermally cured hydrogels. The amount of residual carbon in the PAA hydrogels cured under different conditions was slightly different, and the highest amount of carbon was observed in the hydrogels UV cured for 20 min and thermally cured for 4 h. The higher amount of carbon indicates that the hydrogel is more susceptible to thermal degradation. This also indicates that the hydrogel produces less gaseous matter at high temperatures. The carbon layer formed can cover the surface, providing protection that enhances the heat and ablation resistance [21,22,23]. During the polymerization process, the number of cross-linking points in the PAA hydrogel increased with the curing time, leading to an increase in the cross-linking density. The increase in the cross-link density restricted the movement of the polymer chains, thereby enhancing the interactions between the chain segments. However, a longer curing time can enhance interactions that can make the polymer chains more susceptible to breakage during thermal decomposition.

### 3.5. Solubility, Water Loss Rate, and Hygroscopicity of PAA Hydrogels

**Figure 8:**
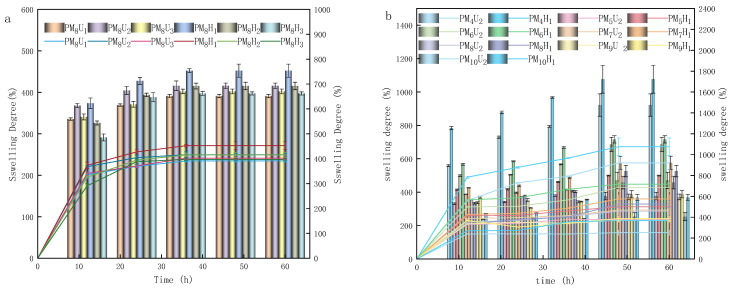
Solubilization of hydrogels formed under different conditions. ((**a**) Different curing conditions; (**b**) different curing conditions and degrees of cross-linking). Lines were added as guidance.

**Figure 9:**
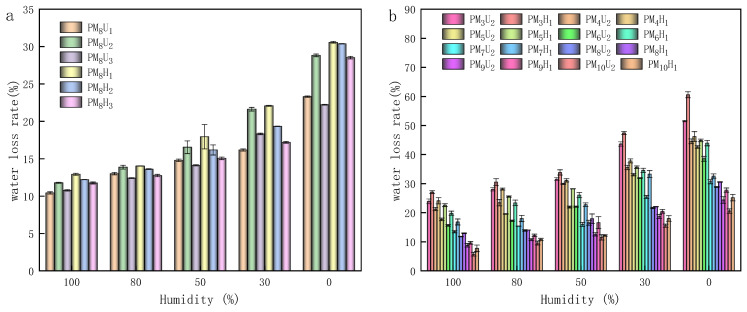
Water loss performance of PAA hydrogel under different conditions: (**a**) the water retention performance of hydrogel formed by different curing methods with a cross-linking agent mass ratio of 3.85%; (**b**) the water retention performance of PAA hydrogel under different conditions.

**Figure 10:**
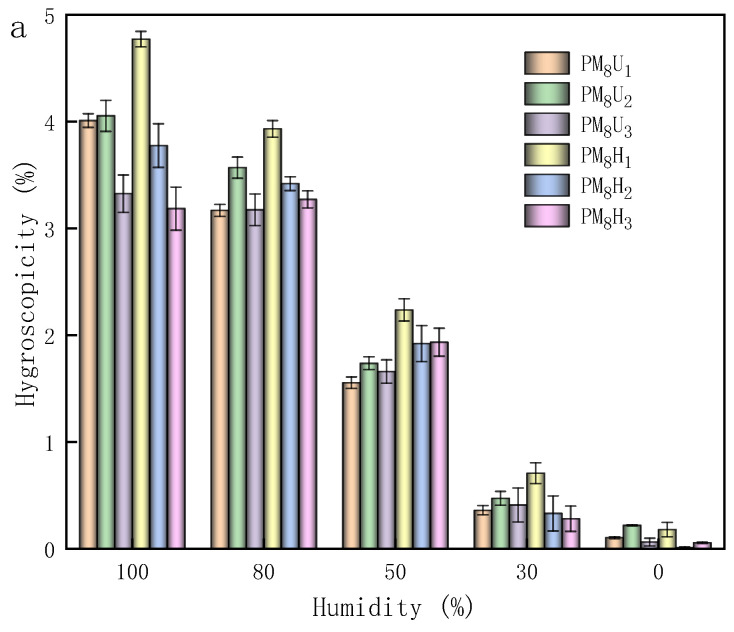

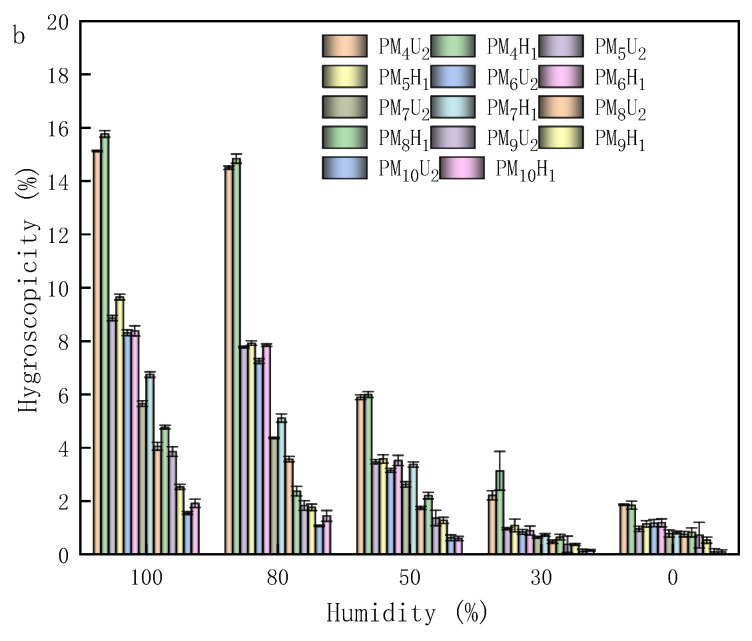
Hygroscopicity of hydrogel formation under different conditions: (**a**) different curing conditions; (**b**) different curing conditions and degrees of cross-linking. Lines were added as guidance.

As shown in Figure 8, during the testing of the swelling properties of the PAA hydrogels, the PAA hydrogels reached the dynamic equilibrium of swelling at 60 h, and the amount of water uptake was stabilized. Figure 8a shows the dynamic equilibrium of the swelling of the UV-cured PAA hydrogels (10, 20, and 30 min) and the thermally cured PAA hydrogels (2, 4, and 6 h) at a cross-linking agent mass ratio of 3.85%. Figure 8b illustrates the equilibrium of swelling plots of the PAA hydrogels cured using UV for 20 min and thermal curing for 2 h at various cross-linking agent mass ratios. As shown in Figure 8a and Figure 9a, the highest swelling and water loss were obtained with UV curing for 20 min and thermal curing for 2 h. The swelling and water loss of the thermally cured hydrogel were slightly higher than those of the UV-cured hydrogel. This phenomenon indicates that the hydrogel has a higher capacity to absorb water molecules and a lower retention capacity to preserve water molecules under these two conditions. However, the increase in the curing time led to a decrease in the swelling degree and water loss rate of the hydrogels. As shown in Figure 8b and Figure 9b, the swelling degree and water loss of the PAA hydrogels significantly decreased with an increasing cross-linking agent mass ratio. This phenomenon could be attributed to the addition of cross-linking agents, which increased the cross-linking density of the polymer chains, thereby reducing the swelling property of the hydrogels. The PAA hydrogels with cross-linking agent mass ratios of 1.96% and 2.91% and that were heat-cured for 2 h exhibited an optimal swelling performance. As shown in Figure 9b, the water loss performance was strongly related to the air humidity. The maximum and minimum water losses of the PAA hydrogels were 27.1% and 5.7% at 100% air humidity, respectively. At 0% air humidity, the maximum and minimum water losses of the PAA hydrogels were 60.6% and 20.6%, respectively. As the air humidity decreased, the hydrogel water loss rate increased. At a constant air humidity, heat curing led to an increased water loss rate, whereas higher degrees of cross-linking decreased the water loss rate. Increasing the cross-linking agent mass ratio of the hydrogel helped to prevent it from losing water in the air environment. As shown in Figure 10a, the moisture absorption rate was the highest under the condition of heat curing for 2 h. However, compared with the other curing methods, the difference in the moisture absorption rate is approximately 1%. Therefore, the difference in the moisture absorption performance and curing method is insignificant. Moreover, the moisture absorption performance and air humidity have a certain relationship. When the air humidity was 100%, the moisture absorption rate was approximately 5%. As shown in Figure 10b, as the cross-linking agent mass ratio increases, the hydrogel moisture absorption performance significantly decreases. Therefore, reducing the cross-linking agent mass ratio decreased the cross-linking density of the hydrogel, which effectively enhanced its ability to absorb water in atmospheric conditions.

### 3.6. Mechanical Properties of Hydrogels

**Figure 11:**
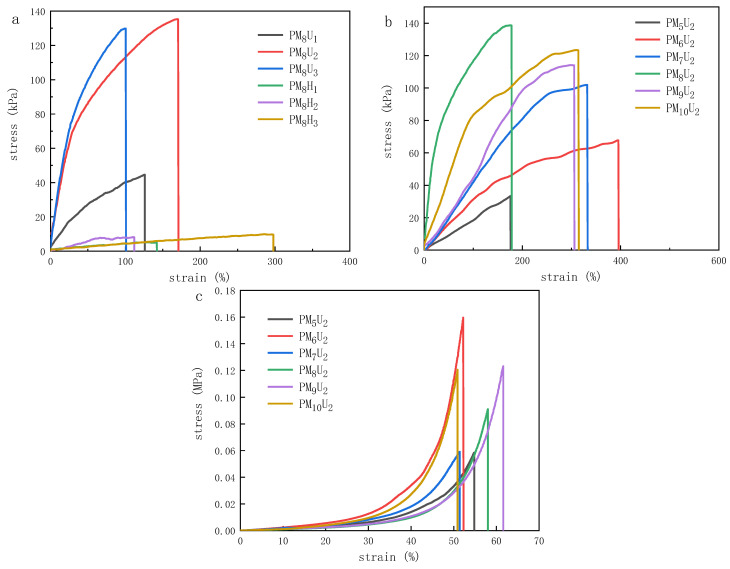
Mechanical properties of hydrogels: (**a**) mechanical tensile properties with different curing methods; (**b**) mechanical tensile properties with different degrees of cross-linking; (**c**) mechanical compression properties with different degrees of cross-linking.

The mechanical tensile test results in Figure 11 and Table 3 reveal that PM_8_U_2_ has the greatest mechanical tensile strength, reaching 135 kPa, followed by PM_10_U_2_ and PM_9_U_2_, which were more than 120 kPa. This variation is because the mechanical tensile properties of the hydrogel are mainly related to the internal formation of the network structure; the more compact it is, the stronger its mechanical properties. PM_5_U_2_ had the largest elongation at break, which was more than 450%. As the degree of cross-linking decreases, the elongation at break increases, resulting in the lower rigidity and greater flexibility of the hydrogel. The mechanical compression test results in Figure 11b reveal that PM_6_U_2_ has the highest mechanical compression strength, at 0.16 MPa. This result shows that the mechanical properties of the polyacrylic acid hydrogel are enhanced as the degree of cross-linking increases. This phenomenon occurs because an increase in the cross-linking agent mass ratio increases the number of connection points between the polymer chains, making the gel more resistant to deformation when subjected to stretching. However, excessive cross-linking can lead to the over-structuring of the polymer chains, potentially reducing their performance. Similarly, an increase in cross-linking improves the mechanical compression properties of the hydrogels. More cross-linking points mean that the gel can better maintain its structural integrity when subjected to compression [24,25,26].

### 3.7. Research on the Application of PAA Hydrogel to Adsorb Lead Ions

**Figure 12:**
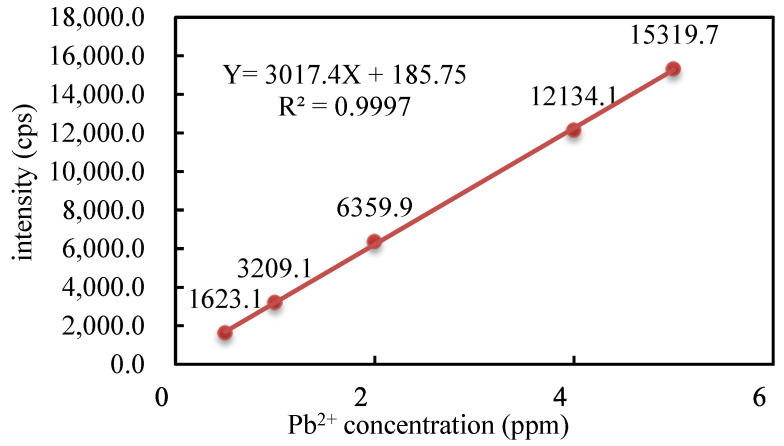
Pb^2+^ standard calibration curve.

The standard curve of Pb^2+^ is plotted in Figure 12. The adsorption capacity of each hydrogel can be calculated using Equation (7). The results shown in Table 4 are as follows: PM_6_U_2_ > PM_6_H_2_, indicating that the particles sometimes accumulated on the pore surface of the thermally cured hydrogels. These particle accumulations might have blocked some of the pores, which made the hydrogels exhibit non-restricted adsorption during the adsorption process, significantly decreasing the adsorption capacity. Except for PM_6_H_2_, the adsorption rates of the thermally cured hydrogels were higher than those of the UV-cured hydrogels. This finding is similar to that of the solubility, hygroscopicity, and water retention performance, which further confirms the important influence of the curing mode on the adsorption properties of hydrogels. This similarity suggests that a high cross-linking agent mass ratio may lead to a smaller pore size of the polymer network, which is one of the key factors affecting the adsorption performance of the hydrogels. The reduced pore size limits the ability of Pb^2+^ to enter the interior of the adsorbent, reducing the adsorption capacity of the adsorbent for heavy metal ions. A moderate degree of cross-linking ensures that the functional groups are uniformly distributed in the polymer network, thus improving its accessibility and adsorption efficiency for heavy metal ions.

## 4. Conclusions

In this study, the mechanical, water absorption, and adsorption properties of hydrogels were significantly influenced by the degree of cross-linking. As the cross-linking agent mass ratio increased, the pore size and volume of the hydrogels decreased, resulting in a more compact structure. This enhancement improved the mechanical properties, reduced the water absorption, and mitigated the water molecule penetration and adsorption behavior. An increase in the cross-linking agent mass ratio increased the number of cross-linking points within the hydrogel, which increased their strength and durability. The hydrogel samples prepared using the UV curing and heat curing methods have different properties. The UV-cured hydrogels with a higher cross-linking agent mass ratio had better mechanical properties, while the heat-cured hydrogels had higher water absorption properties. In addition, the increase in the cross-linking agent mass ratio significantly impacted the mechanical properties of the UV-cured hydrogels, making them suitable for medical applications, such as artificial joints [27], ligaments [28], and tendon repair materials [29]. A lower cross-linking agent mass ratio significantly improved the water absorption properties of the thermally cured hydrogels. In environmental protection [30,31], thermally cured hydrogels with a low cross-linking agent mass ratio can be used to manufacture water-absorbent pads or beads for absorbing and storing spilled oils or hazardous liquids, minimizing environmental pollution. Changes in physicochemical properties can directly affect the application areas and performance of hydrogels. As a result, optimizing the preparation process of polyacrylic acid hydrogels is crucial.

Hence, hydrogel is a versatile material that is increasingly being developed for practical and commercial use. As science and technology continue to advance, hydrogels are expected to play an even more significant role in the future, contributing to both human health and environmental protection.

## Figures and Tables

**Table 1:** Composition and conditions of different polyacrylic acid hydrogels.

Christen	Cross-Linking Agent Mass Ratio (%)	$m_{MBA}$ (g)	Hydrogel Curing Method
PM_3_U_2_	1.48	0.15	UV-20 min
PM_3_H_1_	1.48	0.15	Heat curing-2 h
PM_4_U_2_	1.96	0.20	UV-20 min
PM_4_H_1_	1.96	0.20	Heat curing-2 h
PM_4_H_2_	1.96	0.20	Heat curing-4 h
PM_5_U_2_	2.44	0.25	UV-20 min
PM_5_H_1_	2.44	0.25	Heat curing-2 h
PM_5_H_2_	2.44	0.25	Heat curing-4 h
PM_6_U_2_	2.91	0.30	UV-20 min
PM_6_H_1_	2.91	0.30	Heat curing-2 h
PM_6_H_2_	2.91	0.30	Heat curing-4 h
PM_7_U_2_	3.38	0.35	UV-20 min
PM_8_U_1_	3.85	0.40	UV-10 min
PM_8_U_2_	3.85	0.40	UV-20 min
PM_9_U_2_	4.31	0.45	UV-20 min
PM_9_H_1_	4.31	0.45	Heat curing-2 h
PM_9_H_2_	4.31	0.45	Heat curing-4 h
PM_10_U_2_	4.76	0.50	UV-20 min
PM_10_H_1_	4.76	0.50	Heat curing-2 h
PM_10_H_2_	4.76	0.50	Heat curing-4 h
I-PM_6_U_1_	2.91	0.3 + Photoinitiator I-2959	UV-10 min

**Table 2:** TG test data of different PAA hydrogels.

Sample Name	10% Mass Loss Temperature (°C)	50% Mass Loss Temperature (°C)	Remaining Charcoal (%)
PM_8_H_1_	215.4	384.0	13.3
PM_8_H_2_	227.9	390.1	17.0
PM_8_H_3_	143.7	386.5	14.8
PM_8_U_1_	110.3	385.6	16.6
PM_8_U_2_	100.2	380.8	12.2
PM_8_U_3_	101.1	384.0	14.6
PM_4_H_2_	217.4	390.1	20.5
PM_4_U_2_	209.7	384.0	16.9
PM_10_H_2_	246.0	390.8	14.5
PM_10_U_2_	233.6	392.3	23.2
I-PM_6_U_2_	118.6	380.5	13.6

**Table 3:** Mechanical property data of different PAA hydrogels.

Sample Name	Elastic Modulus (kPa)	Maximum Strength (kPa)	Elongation at Break (%)
PM_8_H_1_	0.05 ± 0.007	5 ± 0.2	142 ± 13
PM_8_H_2_	0.11 ± 0.02	8 ± 0.6	112 ± 12
PM_8_H_3_	0.05 ± 0.003	9 ± 0.6	297 ± 21
PM_8_U_1_	0.78 ± 0.05	45 ± 5	126 ± 14
PM_8_U_2_	2.79 ± 0.14	135 ± 12	171 ± 17
PM_8_U_3_	3.7 ± 0.19	130 ± 11	101 ± 9

**Table 4:** Pb^2+^ adsorption capacity of PAA hydrogel under different conditions.

	PM_5_U_2_	PM_5_H_2_	PM_6_U_2_	PM_6_H_2_	PM_7_U_2_	PM_7_H_2_	PM_8_U_2_	PM_8_H_2_
cps	10,352.5	10,266.4	9948.1	10,084.7	10,337.4	9917.1	10,139.9	9974.3
Pb^2+^ concentration/ppm	3.37	3.34	3.24	3.28	3.36	3.23	3.30	3.24
AC/%	0.42	1.26	4.38	3.04	0.57	4.69	2.50	4.12

## Data Availability

The data presented in this study are available on request from the corresponding author.
